# Exploring *Pediococcus* sp. M21F004 for Biocontrol of Bacterial and Fungal Phytopathogens

**DOI:** 10.3390/md22120534

**Published:** 2024-11-28

**Authors:** Van Thi Nguyen, Yong Min Kwon, Ae Ran Park, Nan Hee Yu, Grace Choi, Jin-Cheol Kim

**Affiliations:** 1 Department of Agricultural Chemistry, Institute of Environmentally Friendly Agriculture, College of Agriculture and Life Sciences, Chonnam National University, Gwangju 61186, Republic of Korea; vanbafu91@gmail.com; 2 Department of Biological Application and Technology, National Marine Biodiversity Institute of Korea, Seocheon 33662, Republic of Korea; jichi9@mabik.re.kr; 3 Plant Healthcare Research Institute, JAN153 Biotech Incorporated, Jeongeup 56212, Republic of Korea; arpark9@naver.com (A.R.P.); nanheeyu0707@gmail.com (N.H.Y.); 4 Department of Biomaterial Research, National Marine Biodiversity Institute of Korea, Seocheon 33662, Republic of Korea

**Keywords:** *Pediococcus* sp. M21F004, oleic acid, antibacterial activity, antifungal activity

## Abstract

This study explores the biocontrol potential of *Pediococcus* sp. M21F004, a lactic acid bacteria (LAB) isolated from marine environments, against several bacterial and fungal phytopathogens. Out of 50 marine bacterial isolates, *Pediococcus* sp. M21F004 was selected for its exceptional antimicrobial activity. The strain, isolated from the intestine of a starry flounder, was identified as *Pediococcus* sp. Gas chromatography–mass spectrometry (GC-MS) analysis revealed that oleic acid (OA) is a key antimicrobial compound produced by *Pediococcus* sp. M21F004. In vitro assays showed that the culture broth (CB) of *Pediococcus* sp. M21F004, as well as OA, exhibited significant inhibitory effects against pathogens such as *Fusarium oxysporum*, *Clarireedia homoeocarpa*, and *Pectobacterium carotovorum* subsp. *carotovorum*. In vivo tests on cucumber *Fusarium* wilt, creeping bentgrass dollar spot, tomato bacterial wilt, and kimchi cabbage soft rot further demonstrated the strain’s efficacy in reducing disease severity. Moreover, OA had the highest control value of 74% against tomato bacterial wilt, followed by 64.1% against cucumber fusarium wilt, 42.5% against kimchi cabbage soft rot, and 16.5% against creeping bentgrass dollar spot. These findings suggest that *Pediococcus* sp. M21F004 and its metabolite OA offer promising alternatives to chemical pesticides, contributing to sustainable plant disease management by promoting resistance induction and providing an eco-friendly approach to agriculture.

## 1. Introduction

Plant diseases caused by bacteria and fungi significantly reduce agricultural production owing to their direct and indirect effects [1]. To manage plant diseases, chemical bactericides and fungicides are employed globally in various agricultural crops [2]. However, these chemicals often exhibit various consequences that adversely affect beneficial soil microorganisms. These consequences lead to the rapid reemergence of soil-borne pathogens, thereby posing a threat to human health and the environment [3,4]. Therefore, developing new and safer bactericides and fungicides is imperative to manage plant diseases effectively. Many biocontrol agents have been recently investigated as alternatives to conventional chemical treatments for managing plant diseases [5,6]. Various bacterial and fungal species demonstrate remarkable antimicrobial properties, proving effective as biocontrol agents against plant diseases [7,8,9,10]. Bacterial control agents dominate these developed biocontrol agents, accounting for approximately 90%, while fungal control agents account for the remaining 10%, because bacteria generally exhibit greater resistance and adaptability as biocides than fungi [11].

Marine microorganisms must survive in extreme conditions, including high pressure, anaerobic environments, low temperatures, high acidity, and ambient temperatures in hot springs. Additionally, they need to adapt to high salinity, radiation, light variations, and reduced nutrients [12]. These extreme conditions in marine environments lead to genetic and metabolic diversity among microorganisms, fostering the development of specific adaptive mechanisms, particularly the synthesis of rare protective compounds. Marine lactic acid bacteria (LAB) are attracting significant interest because of their capacity to release various beneficial compounds, such as bacteriolytic enzymes and organic acids, as well as antimicrobial compounds, such as bacteriocins [13,14,15].

LAB comprise a varied collection of Gram-positive, non-motile, and acid-tolerant microorganisms, which may be rod-shaped or spherical [16]. These bacteria are characterized by their non-spore-forming attributes, the absence of catalase activity, and specific growth requirements, thriving in low pH environments [9,16]. LAB are typically microaerophilic or strictly anaerobic, demonstrating robust adaptability to acidic conditions. Serving as an acidulant, flavor enhancer, preservative, and monomer, LAB are used to produce biodegradable polymer polylactic acid. Additionally, the characteristics of this bacterial strain were investigated for its potential applications in biopesticide formulation. The LAB group includes notable genera such as *Lactobacillus*, *Leuconostoc*, *Pediococcus*, *Lactococcus*, *Enterococcus*, and *Streptococcus* [17].

The biocontrol potential of *Pediococcus* spp. has been explored in recent studies. Isolated from maize leaves, *P. pentosaceus* L006 inhibits *Fusarium proliferatum* and *F. verticillioides* in vitro, effectively suppressing the growth of fumonisin-producing fungi [18]. Furthermore, *P. pentosaceus*, derived from traditional Indian fermented dairy products, demonstrates significant biocontrol potential against the proliferation and zearalenone production of *F. graminearum* [19]. Sixteen LAB strains isolated from various olive oil varieties (*Olea europaea*) exhibit antifungal properties against various pathogens, including *Alternaria*, *Aspergillus*, *Colletotrichum*, *Penicillium*, *Plenodomus*, and *Phytophthora*. Two strains, *Lactiplantibacillus plantarum* and *P. pentosaceus*, exhibit significant inhibitory effects against *F. oxysporum*, various *Colletotrichum* species, and *Penicillium nordicum* [20]. *P. acidilactici* ST3522BG and *P. pentosaceus* ST3633BG, isolated from silage at an eco-friendly farm in Belogradchik, Bulgaria, produced bacteriocins that demonstrate significant antibacterial activity against *Listeria monocytogenes*, *L. innocua*, and *Enterococcus* spp. Additionally, these strains demonstrate antifungal properties against *Alternaria alternata*, *Aspergillus niger*, *Cladosporium sphaerospermum*, *P. chrysogenum*, and *P. expansum* [21]. However, information regarding the effectiveness of antimicrobial metabolites produced by *Pediococci* species in controlling bacterial and fungal plant diseases remains limited, as well as their underlying mechanisms of antimicrobial action.

In this study, from 50 marine bacteria, we selected strain M21F004 with broad-spectrum antimicrobial activity against various phytopathogens. Therefore, this study aims to (1) to screen marine bacterial isolates for antimicrobial activity, followed by the isolation and identification of strain M21F004 as a candidate with significant antimicrobial potential; (2) assess its antimicrobial activity assay against various phytopathogenic bacteria and fungi; and (3) investigate the biocontrol potential of strain M21F004 and its active component, oleic acid (OA), against phytopathogenic diseases caused by bacteria and fungi.

## 2. Results

### 2.1. In Vitro Screening of 50 Marine Bacteria for Antimicrobial Activity

Table 1 shows the 50 bacterial strains that were isolated from marine environments and a list of bacterial strains with general and sample collection information. Among the 50 marine bacterial isolates, only one strain designated as M21F004—isolated from the intestine of a starry flounder (*Platichthys stellatus*)—exhibited significant antibacterial activity against *Agrobacterium tumefaciens*, *Ralstonia solanacearum* SL341, and *Xanthomonas arboricola* pv. *pruni* with the same minimum inhibitory concentration (MIC) values of 10% of cell-free supernatant (CFS). Furthermore, it showed significant antifungal activity against *Rhizoctonia solani*, *Clarireedia homoeocarpa*, and *Pythium aphanidermatum* with MIC values of 10%, 10%, and 5% of CFS, respectively (Tables S1 and S2). Therefore, this strain was selected and examined further. Its optimum culture conditions, including temperature, pH, and NaCl concentration, were determined for further experiments. Growth of strain M21F004 was observed at 15–30 °C (optimum, 25 °C), pH 4–10 (optimum, pH 7), and in the presence of 0–6% (*w*/*v*) NaCl (optimum, 1%).

### 2.2. Identification of Strain M21F004

**Figure 1:**
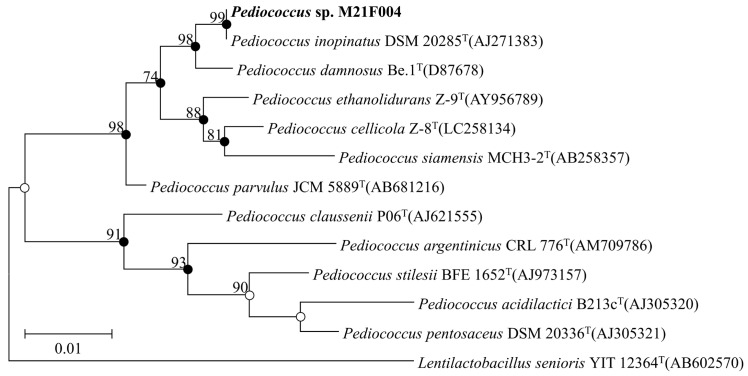
ML phylogenetic tree based on 16S rRNA gene sequences, showing the relationships between strain M21F004 (highlighted in bold) and closely related taxa with validly published names. GenBank accession numbers are provided in parentheses. Bootstrap values (>70%) are shown at nodes as percentages based on 1000 replicates. Closed and open circles represent nodes supported via all three treeing methods (ML, MP, and NJ) or through two treeing methods, respectively. *Lentilactobacillus senioris* YIT 12364^T^ was used as the outgroup. Scale bar represents 0.01 changes per nucleotide position. ML, maximum likelihood; MP, maximum parsimony; NJ, neighbor-joining.

Comparative 16S rRNA gene sequence analysis showed that strain M21F004 was closely related to *Pediococcus inopinatus* DSM 20285^T^, sharing 100% similarity. Phylogenetic analysis based on 16S rRNA gene sequences using the maximum-likelihood (ML), maximum-parsimony (MP), and neighbor-joining (NJ) algorithms further confirmed that strain M21F004 formed a monophyletic clade with other species in the genus *Pediococcus* (Figure 1). Consequently, based on the nearly complete 16S rRNA gene sequence comparison, the strain was clearly affiliated with the isolated genus *Pediococcus* and was consequently named *Pediococcus* sp.

### 2.3. Histochemical β-Glucuronidase Protein Expression by Induced Resistance of Strain M21F004

**Figure 2:**
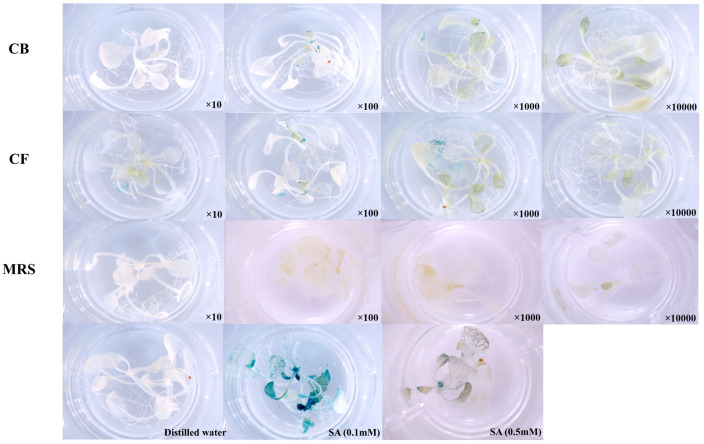
GUS expression in PR1::GUS *Arabidopsis* seedlings activated by treatment with M21F004 CB. GUS, histochemical β-glucuronidase; CB, culture broth of strain M21F004; CFS, cell-free supernatant of strain M21F004; MRS, de Man, Rogosa, and Sharpe as a negative control.

Treatment with 1000-fold and 10,000-fold dilutions of the culture broth (CB) and CFS from strain M21F004 resulted in β-glucuronidase (GUS) protein expression comparable to that observed in the salicylic acid (SA) control group. Conversely, plants treated solely with the medium, serving as a negative control, did not show any GUS protein expression (Figure 2). Therefore, these findings indicate that strain M21F004 has potential bioactive compounds capable of inducing certain plant responses similar to those induced by SA.

### 2.4. Identification of Antimicrobial Metabolites

The components isolated from the hexane (Hex)-extracted layer of *Pediococcus* sp. M21F004 are shown in Table 2 and Figure S1. The results show six different peaks in the Hex-extracted layer, accounting for 85–95% of the relative composition. OA was identified as the predominant component in this layer, comprising 39.9% with a retention time of 10.69 min. This was similar to the OA standard with a retention time of 10.75 min (Table 2 and Figure S1). The similarity in retention times and high percentage confirm OA as the main bioactive compound in the Hex-extracted layer. Future studies should use OA to confirm its antimicrobial activity.

### 2.5. Efficacy of Pediococcus sp. M21F004 Culture Broth and OA in Controlling Cucumber Fusarium Wilt

**Figure 3:**
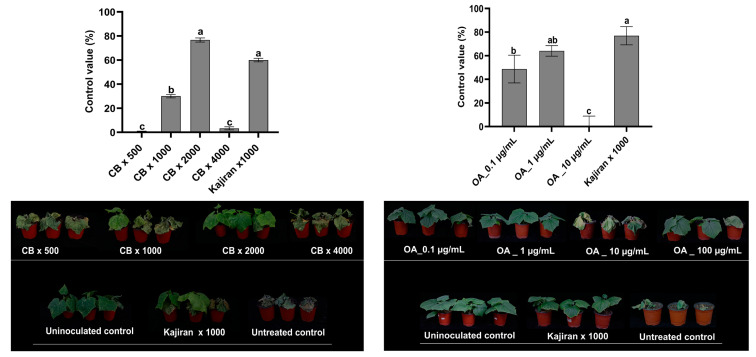
Efficacy of *Pediococcus* sp. M21F004 CB in controlling cucumber *Fusarium* wilt. The control group (100%) represents disease severity in untreated cucumber plants, with efficacy expressed as the percentage reduction in disease severity relative to this control. Values are presented as mean ± standard error from three independent trials, each with nine replicates. Bars sharing the same letters indicate non-significant differences among treatments (*p* < 0.05, Fisher’s LSD test). CB, culture broth of *Pediococcus* sp. M21F004; OA, oleic acid; LSD, least significant difference.

Figure 3 shows the results of the efficacy of *Pediococcus* sp. M21F004 CB and OA in managing *Fusarium* wilt in cucumbers when evaluated across various dilutions and concentrations. Treatment with *Pediococcus* sp. M21F004 CB at a 2000-fold dilution showed the highest efficacy in managing cucumber *Fusarium* wilt, achieving a 76.7% reduction in *Fusarium* growth compared to the untreated control. This efficacy was statistically similar to that of the commercial product Kajiran, which showed a control value of 60% at a 1000-fold dilution. However, when the concentration of *Pediococcus* sp. M21F004 CB exceeded the 2000-fold dilution, and a decrease in control value was observed.

Similarly, OA exhibited the highest control value of 64.1% at a concentration of 1 µg/mL, which was statistically similar to that of a commercial standard Kajiran (76.9%). However, at a higher concentration of 10 µg/mL, OA did not demonstrate significant control efficacy. In the in vivo experiments of *Fusarium* wilt in cucumbers, the disease control efficacy did not show concentration-dependent response to *Pediococcus* sp. M21F004 CB and OA. The reason why CB *Pediococcus* sp. M21F004 CB and OA show high disease control efficacy at low concentrations may be less pronounced due to biological diversity and complex interactions between plants and pathogens under in vivo conditions but may be due to the characteristic of induced resistance, showing high activity at low concentrations.

### 2.6. Efficacy of Pediococcus sp. M21F004 Culture Broth and OA in Controlling Creeping Bentgrass Dollar Spot

**Figure 4:**
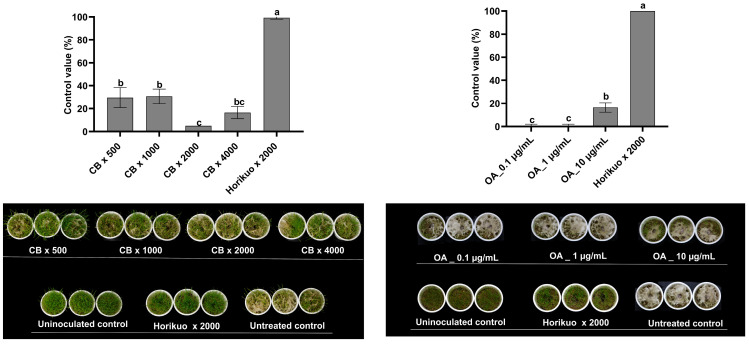
Efficacy of *Pediococcus* sp. M21F004 CB in controlling creeping bentgrass dollar spot. The control group (100%) represents disease severity in untreated creeping bentgrass, with efficacy expressed as the percentage reduction in disease severity relative to this control. Values are presented as mean ± standard error from three independent trials, each with nine replicates. Bars sharing the same letters indicate non-significant differences among treatments (*p* < 0.05, Fisher’s LSD test). CB, culture broth of *Pediococcus* sp. M21F004; OA, oleic acid; LSD, least significant difference.

The effects of *Pediococcus* sp. M21F004 CB and OA in suppressing dollar spot caused by *C. homoeocarpa* in creeping bentgrass were investigated (Figure 4). Treatment with M21F004 CB at 500-fold and 1000-fold dilutions resulted in control values of 29.6% and 30.7%, respectively. These values were lower than the control value of 99% achieved by a commercial standard Horikuo (25% Tebuconazole) at a 2000-fold dilution. Similarly, OA treatment at 10 µg/mL yielded a control value of 16.5%, which is lower than that of Horikuo.

### 2.7. Efficacy of Pediococcus sp. M21F004 Culture Broth and OA in Controlling Tomato Bacterial Wilt

**Figure 5:**
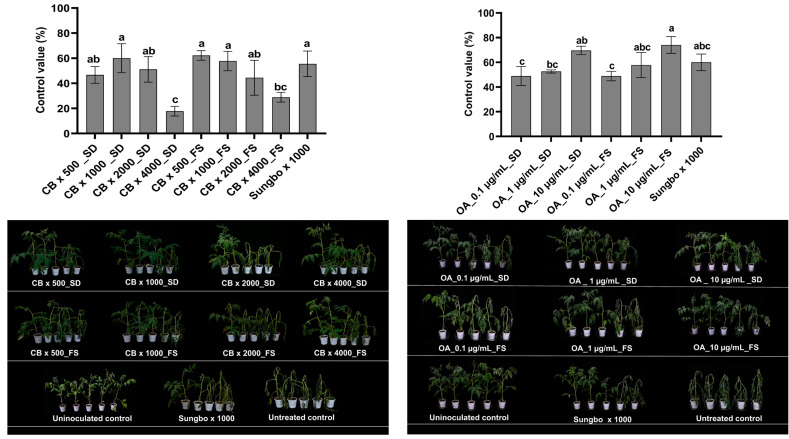
Efficacy of *Pediococcus* sp. M21F004 CB in controlling tomato bacterial wilt. The control group (100%) represents disease severity in untreated tomato plants, with efficacy expressed as the percentage reduction in disease severity relative to this control. Values are presented as mean ± standard error from three independent trials, each with nine replicates. Bars sharing the same letters indicate non-significant differences among treatments (*p* < 0.05, Fisher’s LSD test). CB, culture broth of *Pediococcus* sp. M21F004; OA, oleic acid; SD, soil drench; FS, foliar spray; Sungbo, Sungbocycline (×1000); LSD, least significant difference.

The effectiveness of *Pediococcus* sp. M21F004 CB and OA against tomato bacterial wilt were quantitatively evaluated using various concentrations and treatment methods (Figure 5). *Pediococcus* sp. M21F004 CB exhibited high control values of 62.2% and 57.8% when applied as foliar sprays at 500-fold and 1000-fold dilutions, respectively. When applied as a soil drench, the 1000-fold dilution achieved a high control value of 60%. These values were statistically similar to the 55.6% control value observed with the commercial antibacterial agent Sungbocycline.

Similarly, the disease control efficacy of OA was evaluated across concentrations ranging from 0.1 to 10 µg/mL, applied as soil drenches and foliar sprays. OA exhibited a dose-dependent increase in efficacy. When treated as a foliar spray, it achieved significant control values of 57.8% and 74% at concentrations of 1 and 10 µg/mL, respectively. Conversely, when treated as a soil drench, it achieved a control value of 69.6% at 10 µg/mL concentration. These control levels were statistically similar to that of the commercial antibacterial agent Sungbocycline, which showed a control value of 60%. No significant difference was observed in the disease control effectiveness of *Pediococcus* sp. M21F004 CB and OA against tomato bacterial wilt, depending on treatment methods employed. None of the samples exhibited any phytotoxic symptoms at the tested concentrations.

### 2.8. Efficacy of Pediococcus sp. M21F004 Culture Broth and OA in Controlling Kimchi Cabbage Soft Rot

**Figure 6:**
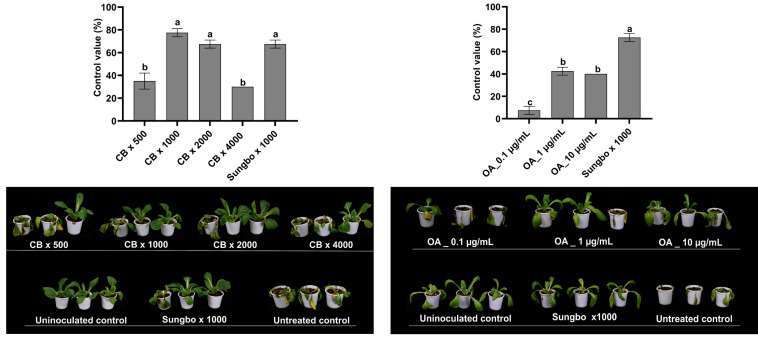
Efficacy of *Pediococcus* sp. M21F004 CB in controlling kimchi cabbage soft rot. The control group (100%) represents disease severity in untreated kimchi cabbage, with efficacy expressed as the percentage reduction in disease severity relative to this control. Values are presented as mean ± standard error from three independent trials, each with nine replicates. Bars sharing the same letters indicate non-significant differences among treatments (*p* < 0.05, Fisher’s LSD test). CB, culture broth of *Pediococcus* sp. M21F004; OA, oleic acid; Sungbo, Sungbocycline (×1000); LSD, least significant difference.

The effects of *Pediococcus* sp. M21F004 CB and OA in suppressing soft rot caused by *Pectobacterium carotovorum* subsp. *carotovorum* in kimchi cabbage was investigated (Figure 6). The control value against kimchi cabbage soft rot was 77.5% and 67.5% when treated with 1000-fold and 2000-fold dilutions of the M21F004 CB, respectively. These values were statistically similar to that of the commercial antibacterial agent Sungbocycline, which was 67.5%.

In parallel, OA demonstrated control values of 42.6 and 40% against kimchi cabbage soft rot when treated at concentrations of 1 and 10 µg/mL, respectively.

## 3. Discussion

In this study, 50 marine bacterial strains were screened, and strain M21F004 was selected owing to its significant antimicrobial activities and capacity to induce resistance in in vitro and in vivo systems. *Pediococcus* sp. M21F004—used in this study—belongs to the family *Lactobacillaceae*. This family encompasses various LAB species and is one of the most prominent probiotic groups [22]. Although most LAB species are non-pathogenic, they can occasionally cause opportunistic infections in humans. Nonetheless, their long history of safe use leads to their classification under the qualified presumption of safety status [23,24]. This study presents the first report of strain M21F004 CB exhibiting efficacy against tomato bacterial wilt caused by *R. solanacearum* SL341, soft rot disease of kimchi cabbage caused by *P. carotovorum* subsp. *carotovorum*, *Fusarium* wilt disease of cucumber caused by *Fusarium oxysporum* f. sp. *cucumerinum*, and dollar spot of creeping bentgrass by *C. homoeocarpa* in planta. Previous research demonstrates that *P. pentosaceus* exhibits significant antifungal activity, effectively inhibiting the growth of *P. expansum*, a mold responsible for apple rot, by producing antifungal peptides [25]. This suggests potential applications for such strains in the food industry to prevent fungal spoilage [25]. Furthermore, several *P. pentosaceus* strains PP-601, FB-301, FG-401, CR-501, PP-402, and CB-402 exhibit significant antifungal activity against a spectrum of fungi, including *F. graminearum*, *Rhizopus stolonifer*, *Sclerotium oryzae*, *R. solani*, *Botrytis cinerea*, and *Sclerotinia minor*. This makes them promising candidates for biopreservation applications aimed at extending the shelf-life of fresh vegetables [26]. Building on these findings, we concluded that *Pediococcus* sp. M21F004 could potentially exhibit antibacterial and antifungal activity against various phytopathogenic bacteria and fungi.

An active compound with antibacterial and antifungal activity was isolated and identified from this strain. In this study, *Pediococcus* sp. M21F004 was found to produce OA as its antimicrobial metabolite, identified through GC-MS with a 95% similarity to the OA in the GC-MS library, and further confirmed by matching the retention time of 10.75 min with an OA standard. OA is commonly found in plants and animals, serving as a natural raw material with minimal environmental influence [27]. OA is identified as the third most prevalent component of rose essential oil, as detected via GC-MS [28]. GC-MS analysis further reveals that OA and palmitic acid are the predominant active compounds in the CCl_4_ fraction of sesame cake crude extracts. Both compounds, derived from sesame seed cake extracts, significantly enhance cucumber plant growth and chlorophyll content while reducing sodium levels in shoots. Furthermore, they significantly promote soil microbial activity, particularly bacterial populations, suggesting their potential for improving soilborne disease resistance in greenhouse and orchard environments [29].

OA exhibits significant antimicrobial potential in various applications, including its activity against human pathogens. Previous studies indicate that OA demonstrates antimicrobial activity against three Gram-positive bacteria with a MIC of 1.0 mg/mL, including *Bacillus subtilis*, *Micrococcus kristinae*, and *Staphylococcus aureus* [30]. Additionally, Chandrasekaran et al. [31] report that hexadecanoic acid and OA exhibit antifungal and antibacterial activities against *B. subtilis*, *Micrococcus luteus*, *S. aureus*, *B. pumilus*, *Klebsiella pneumoniae*, *Pseudomonas aeruginosa*, and *Candida albicans*. The antimicrobial activity of fatty acids—including myristic acid, lauric acid, capric acid, palmitoleic acid, and OA—is effective against tuberculosis-causing pathogens [32]. Kabara et al. [33] found that lauric acid, OA, and linoleic acid exhibit biocidal properties against Gram-positive bacteria. Additionally, Zhu et al. [34] report that OA significantly inhibits the growth of *C. albicans* and *A. fumigatus*. Another study reports that methyl ferulate and OA are metabolites produced by *A. pseudodeflectus* AU MC 15761. OA and methyl ferulate exhibit significant antioxidant and antibacterial activities, with OA demonstrating MIC values of 1.25 mg/mL against *B. subtilis* and 0.62 mg/mL against *S. aureus*. Both compounds have been reported to have antioxidant activity (as measured by the percentage of Diphenylpicrylhydrazyl scavenging) and effectively bind to key proteins in *S. aureus* [35]. However, studies on OA activity against phytopathogenic diseases in plants remain few. OA significantly inhibits the mycelial growth of *Pyrenophora ultimum* and *Crinipellis perniciosa*, whereas *R. solani* and *P. avenae* show no significant changes in mycelial growth. It significantly reduces the mycelial growth of *Pythium ultimum* at 100 μM concentration and *C. perniciosa* at 1000 μM concentration [36]. Metabolites—including pentadecanoic acid ethyl ester, palmitoleic acid, n-hexadecanoic acid, hexadecanoic acid ethyl ester, OA, linoleic acid ethyl ester, ethyl oleate, and ergosterol derived from *Trichoderma asperellum* and *T. longibrachiatum*—exhibit antifungal activity against *F. xylarioides* [37]. A previous study reported that OA, at 0, 100, 1000, 2000, and 3200 μmol/L concentrations, does not inhibit the mycelial growth of *Alternaria solani*, *Cladosporium lagenarium*, *F. oxysporum* f. sp. *cucumerinum*, and *F. oxysporum* f. sp. *lycopersici*. Additionally, these concentrations, up to 3200 μmol/L, do not inhibit the spore germination of *C. lagenarium*, *F. oxysporum* f. sp. *cucumerinum*, and *F. oxysporum* f. sp*. lycopersici* [38]. Our study is the first to report the activity of OA against tomato bacterial wilt caused by *R. solanacearum* SL341, soft rot disease in kimchi cabbage caused by *P. carotovorum* subsp. *carotovorum*, *Fusarium* wilt disease in cucumber caused by *F. oxysporum* f. sp. *cucumerinum*, and dollar spot in creeping bentgrass caused by *C. homoeocarpa* in planta.

*Pediococcus* sp. strain M21F004, one of the most prominent probiotic groups, has significant antimicrobial activities and capacity to induce resistance in in vitro and in vivo systems. In particular, our findings suggest that *Pediococcus* sp. M21F004 CB can effectively induce resistance in transgenic *Arabidopsis* plants expressing *PR1pro::GUS* and control the progression of *R. solanacearum* SL341, soft rot disease in kimchi cabbage, and *Fusarium* wilt disease in cucumber, even when treated at low concentrations seven and three days before inoculation, compared to that of the positive control. *PR1* gene is a marker gene of systemic defense in plants. Therefore, OA may be a key factor in the resistance induced in plants following treatment with *Pediococcus* sp. M21F004 CB. Additionally, other compounds, such as 3-Isobutylhexahydropyrrolo [1,2-a] pyrazine-1,4-dione, palmitic acid, and 3-Benzyl-hexahydro-pyrrolo [1,2-a] pyrazine-1,4-dione, may also contribute to the induced resistance observed. These active compounds could potentially enhance systemic defense and promote growth in various plants, leading to healthier and more resistant plants, as well as fostering healthy soil microbial communities. Therefore, further research is needed to identify all plant resistance inducers present in the CB and CFS of strain M21F004.

OA demonstrated the highest control value (74.1%) against tomato bacterial wilt, followed by the control value against cucumber *Fusarium* wilt disease (64.1%). The lower control values observed for kimchi cabbage soft rot (42.6%) and creeping bentgrass dollar spot (16.5%) suggest that the efficacy of OA varies depending on the specific plant and pathogen involved. These differences in control values can be attributed to the unique interactions between each host plant and its corresponding pathogen, as well as the varying susceptibility of different pathogens to OA. Taken together, *Pediococcus* sp. M21F004 was identified as a novel biocontrol agent among 50 marine bacteria because of its significant antifungal and antibacterial activities against several pathogens, as well as its ability to induce resistance in plants. Furthermore, our findings highlight the biocontrol potential of OA against phytopathogens, suggesting its wider applicability in plant disease management. This potential highlights the versatility of OA as a promising antimicrobial agent for agricultural applications.

## 4. Materials and Methods

### 4.1. Marine Bacterial Isolation and Culture Conditions

The bacterial strains used in this study were isolated from marine environments using the following procedure. Samples were collected from marine algae (*Codium fragile*, *Undaria pinnatifida*, and *Ulva* sp.), a plant (*Rumex crispus*), the intestine of a fish (*P. stellatus*), sediments, and seawater. The marine sediments and seawater samples were diluted with sterile 0.85% saline. The intestinal tissue of a fish purchased from a general fish market was homogenized and similarly diluted with sterile saline. Additionally, small pieces of algal fronds and plant roots were homogenized and diluted with sterile saline. The serially diluted samples, ranging from 10^−1^ to 10^−3^, were spread on MRS (Merck) agar containing bromocresol purple (Merck), marine agar 2216 (BD), R2A agar (BD), and nutrient agar (BD) plates. The inoculated plates were incubated at 28 °C for 5 days. Individual colonies—selected based on morphological difference or the presence of yellowish zones (indicative of LAB)—were then selectively isolated on the agar mentioned above media. Following primary isolation and purification, the strains were routinely cultured on each agar media at 28 °C and preserved in 20% (*v*/*v*) glycerol at −80 °C.

The optimum culture condition for the selected LAB strain M21F004 was assessed in MRS broth by measuring the optical density at 600 nm (OD_600_) using a personal bioreactor (RTS-8, BioSan, Riga, Latvia) for 3 days. Growth conditions were assessed across various conditions, including temperature (15–50 °C, at 5 °C intervals), pH (2–10, at one-unit intervals, adjusted with 1M NaOH or HCl), and NaCl concentrations (0–10%, at 1% intervals, *w*/*v*).

### 4.2. Preparation of CFS from Marine Bacterial Strains

The fifty marine bacterial strains used in this study were cultured in 50 mL of the respective liquid broths, as previously described, at 28 °C for 2–3 days. The cells were separated via centrifugation at 7000 rpm, 4 °C for 10 min, and they were subsequently removed. The supernatants were then filter-sterilized using a 0.22 µm pore size syringe filter (Corning, New York, NY, USA). These filter-sterilized supernatants, designated as CFS of marine bacterial strains, were used for MIC testing and stored at −20 °C until required for further assays.

### 4.3. Phytopathogenic Microorganisms and Culture Conditions

In this study, four pathogenic bacteria and four pathogenic fungi were used for MIC testing. The bacterial pathogens included *A. tumefaciens* (crown gall of apple), *P. carotovorum* subsp. *carotovorum* (bacterial soft rot of kimchi cabbage), *X. arboricola* pv. *pruni* (bacterial spot of stone fruit), and *R. solanacearum* SL341 (bacterial wilt of tomato). The fungal pathogens used were *C. homoeocarpa* (dollar spot of creeping bentgrass), *R. solani* (sheath blight of rice), *Phytophthora infestans* (late blight of tomato), and *P. aphanidermatum* (damping-off).

The pathogenic bacteria were cultured on Tryptic Soy Agar (TSA) and in Tryptic Soy Broth (TSB) media at 30 °C, except for *X. arboricola* pv. *pruni*, which required a lower incubation at 28 °C. All pathogenic fungi were cultured on potato dextrose agar (PDA) at 25 °C, except for *P. infestans*, which was cultured on V8 juice agar. Broth cultures of all fungal pathogens were prepared in potato dextrose broth (PDB) and incubated at 25 °C. All media, TSA, TSB, PDA, and PDB, were supplied by Becton, Dickinson, and Company, Sparks, MD, USA.

### 4.4. In Vitro Screening of Antimicrobial Activities

The MIC values of 50 marine bacteria against various test microorganisms were determined using the micro-broth dilution method to identify potential biocontrol agents. This assay was conducted in a 96-well sterile microplate, following the procedures outlined in previous studies [6]. The CFS concentration used in this study ranged from 0.16 to 10%, adjusted through serial two-fold dilutions. Streptomycin sulfate (Sigma-Aldrich Co., St. Louis, MO, USA) served as the positive control. Pathogens treated with uninoculated growth media served as the negative control. The test bacteria were incubated at 30 °C for 1–2 days, whereas the test fungi were incubated at 25 °C for 3–7 days. Bacterial suspensions were prepared by culturing in TSB until the mid-log phase and adjusting the optical density to OD_600_ = 0.1, corresponding to approximately 1 × 10⁸ CFU/mL. Fungal suspensions were prepared by culturing on PDA, harvesting conidia using sterile distilled water containing 0.01% Tween 80, and counting spores using a Neubauer chamber. The spore suspensions were adjusted to 1 × 10⁵ spores/mL. For each assay, 50 μL of CFS was combined with 50 μL of the pathogen suspension in each well, resulting in a final volume of 100 μL per well. For fungal assays, optical density measurements were taken at OD_600_ Wells exhibiting extensive mycelial growth were visually inspected to account for turbidity limitations in spectrophotometric readings. The MIC was defined as the lowest concentration capable of inhibiting microbial growth. Bacterial and fungal growth was assessed by measuring the OD_600 nm_. Each experiment was repeated twice, with six replicates each time.

### 4.5. Phylogenetic Analysis Based on 16S rRNA Sequences

Genomic DNA from strain *Pediococcus* sp. M21F004, selected after initial screening, was extracted using an Exgene DNA extraction kit (GeneAll, Seoul, Republic of Korea), following the manufacturer’s instructions. The 16S rRNA gene was amplified via PCR using bacteria-specific universal primers 27F and 1492R [39]. The amplified partial 16S rRNA gene was sequenced using an Applied Biosystems automated sequencer (ABI 3730XL) at Macrogen Co., Ltd. (Seoul, Republic of Korea) and assembled into a nearly full-length 16S rRNA gene sequence using Geneious program v9.0.5. The phylogenetic position of strain M21F004 was identified by searching the EzBioCloud server (ezbiocloud.net/identify) [40], whereas the 16S rRNA gene sequence (1529 nucleotides) was compared with sequences of validly published species in the server. The 16S rRNA gene sequence has been deposited in GenBank under accession number PQ136537. Phylogenetic trees, based on 1424 unambiguously aligned sequences, were reconstructed using the ML, MP, and NJ algorithms [41,42,43] in MEGA X [44]. To assess the robustness of the tree topologies, 1000 bootstrap resampling datasets [45] were conducted for each algorithm.

### 4.6. Histochemical β-Glucuronidase Staining Assay

To investigate pathogenesis-related gene 1 (*PR1*) expression, a marker of induced resistance, we used transgenic *Arabidopsis thaliana* plants carrying a *GUS* reporter gene fused with the *PR1 promoter*. The seeds were first sterilized by immersing in 70% ethanol for 3 min, followed by 100% ethanol for 1 min, and subsequently rinsed once with sterile distilled water. The sterilized seeds were subsequently sown on half-strength Murashige–Skoog medium (comprising 2.2 g MS salts, 10 g sucrose, and 8 g phyto agar per liter) supplemented with 50 μg/mL kanamycin [46]. These seeds were cultured in a growth chamber under a 16 h photoperiod at 25 °C and 80% relative humidity (RH) for 12 days.

Two 12-day-old *A. thaliana* plants were transplanted into each well of a 24-well plate, with each well containing 2.5 mL of liquid medium mixed with the CB and CFS of *Pediococcus* sp. M21F004 at final dilutions of 10-fold, 100-fold, 1000-fold, and 10,000-fold. In this study, the CB refers to the whole culture containing both bacterial cells and extracellular metabolites. The CFS refers to the filtered culture broth, which excludes bacterial cells but retains extracellular metabolites. The plants were subsequently incubated at room temperature on an orbital shaker for 2 days. Salicylic acid served as a positive control, whereas MRS medium served as a negative control. Histochemical GUS staining was performed as described previously [47]. GUS activity was qualitatively assessed by observing the development of a blue color under an optical microscope (Stemi 508; Carl Zeiss, Jena, Germany).

### 4.7. Extraction and Structural Determination of the Active Metabolite from Strain M21F004

The *Pediococcus* sp. M21F004 was cultured on MRS agar at 25 °C for 3 days. The strain M21F004 was subsequently incubated in MRS broth on a rotary shaker at 150 rpm, 25 °C for 3 days, then filtered through four layers of cheesecloth. The supernatant (200 mL) from the CB was then successively partitioned with equal volumes of Hex. The Hex layers were combined and concentrated using a rotary evaporator at 40 °C to remove any residual organic solvent.

The Hex-extracted layer (200 µL, 0.1 µg/mL), which was mixed with 1 mL of Silyl-991 (BSTFA 95–100%, CAS 25561-30-2), was analyzed using GC-MS (Shimadzu GC-MS QP5050, Shimadzu Co., Kyoto, Japan). Analytes were separated on an Agilent Technologies DB-5MS column (30 m × 0.25 mm × 0.25 µm). Helium was used as the carrier gas at a flow rate of 1.22 mL/min. The column oven temperature program commenced at 30 °C, increased to 280 °C at 30 °C/min, then to 300 °C at 5 °C/min, and was maintained for 2 min. The total runtime was 40 min. The injection port temperature was maintained at 300 °C, with an injection volume of 150 µL in splitless mode. Mass spectra from the Hex-extracted layers were compared with those in the NIST/EPA/NIH Mass Spectral Library (version 2.0). Quantities of the Hex-extracted layer were expressed as relative area concentrations, adjusted to account for the areas of compounds identified in the negative controls.

### 4.8. In Vivo Antimicrobial Activities

#### 4.8.1. Fusarium Wilt of Cucumber

The assay was conducted on cucumber plants at the two-true-leaf stage, following the method described by Lee et al. [48]. Cucumber seeds—cultivar ‘Chungboksamchok’, Syngenta, South Korea—were first germinated on moist tissue paper in a Petri dish at 28 °C for approximately 24 h. The pathogen, *F. oxysporum* f. sp. *cucumerinum*, was prepared with the following steps. *F. oxysporum* f. sp. *cucumerinum* was sub-cultured onto PDA plates and incubated at 25 °C for 5 days. Millet seeds (50 g) were soaked overnight in 100 mL of distilled water in a 250 mL flask. After soaking, the water was discarded, and the seeds were autoclaved at 121 °C for 20 min on 2 consecutive days. Conidia were harvested from 5-day-old *F. oxysporum* f. sp. *cucumerinum* cultures grown on solid medium using sterile distilled water containing 0.01% Tween 80. The conidial concentration was quantified using a Neubauer chamber and adjusted to 1 × 10⁶ conidia/mL to ensure consistent inoculation. This standardized conidial suspension was used to inoculate the millet seed medium. The flask was then incubated at 25 °C for 2 weeks, with shaking every 2 days to promote uniform colonization. After incubation, the *F. oxysporum* f. sp. *cucumerinum* culture was sieved through a 3 mm mesh. The resulting conidial suspension was used directly in the assays, maintaining the concentration at 1 × 10⁶ conidia/mL to achieve a 0.5% (*w*/*v*) pathogen concentration.

The germinated seeds were then transplanted into vinyl pots (5.5 cm in diameter, 6 cm in height) filled with nursery soil and inoculated with 0.5% (*w*/*v*) of the pathogen *F. oxysporum* f. sp. *cucumerinum.* The seedlings were subsequently grown at 25 ± 5 °C and 77 ± 5% RH under a 16 h light/8 h dark cycle. Seven and three days before inoculation, the plants were treated with 20 mL of soil drench per pot using dilutions of M21F004 CB medium at 500-fold, 1000-fold, 2000-fold, and 4000-fold or with OA at concentrations of 0.1, 1, and 10 µg/mL. Oleic acid used in this study was purchased from Sigma-Aldrich (St. Louis, MO, USA) to ensure a standardized and reliable comparison in evaluating its effects on pathogen inhibition. Each dilution was supplemented with Tween 20 at a concentration of 250 µg/mL to ensure consistency in surfactant conditions across all treatments. Kajiran (etridiazole 10% + thiophanate-methyl 55% WP; Hankook Samgong Co., Seoul, Korea) was used as the positive control, whereas a Tween 20 without CB served as the negative control. No significant effect on disease severity was observed from the application of Tween 20 alone. Disease severity (DS) was assessed 2 weeks after inoculation using a scale where 0 indicated no symptoms; 1 indicated < 25% of leaves with yellowing or necrosis; 2 indicated 26–50% of leaves affected; 3 indicated 51–75% of leaves affected; and 4 indicated 76–100% of leaves showing wilt, yellowing, and/or necrosis [49]. The experiments were conducted twice with twelve replicates each.

#### 4.8.2. Dollar Spot of Creeping Bentgrass

Creeping bentgrass seeds were soaked in distilled water at 4 °C for 48 h. After incubation, they were transferred to vinyl pots (5.5 cm in diameter, 6 cm in height), followed by cultivation at 25 ± 3 °C with 95 ± 5% RH under a 16 h light/8 h dark cycle for 3 weeks. Seven and three days before inoculation, the plants were treated with a soil drench application of 20 mL per pot using M21F004 CB medium diluted 500-fold, 1000-fold, 2000-fold, and 4000-fold or OA at concentrations of 0.1, 1, and 10 µg/mL. Additionally, each treatment included Tween 20 at a concentration of 250 µg/mL. The pathogen *C. homoeocarpa* was cultured on a PDA medium for 7 days, and it was then cut into 1 cm^2^ pieces. Five of these pieces were then inoculated onto a medium consisting of 9 g of wheat bran, 1.5 g of rice husk, and 10 mL of distilled water. The medium was autoclaved twice, with an additional 10 mL of distilled water added after the first autoclaving. The inoculum was then incubated at 25 °C for 7 days. After 10 days of incubation, 110 mL of sterilized water was added to the medium and the mixture was blended. Subsequently, 3.5 mL of the resulting suspension was used to inoculate the soil in each pot. The treated plants were then inoculated with 3.5 mL of *C. homoeocarpa* inoculum. DS was assessed 7 days after inoculation by measuring the extent of leaf yellowing in the turfgrass. A 2000-fold dilution of Horikuo (25% Tebuconazole) served as the positive control. The experiments were conducted twice in nine replicates each.

#### 4.8.3. Bacterial Wilt of Tomato

Tomato seeds (cultivar ‘Seokwnang’; FarmHannong Co., Ltd., Seoul, Republic of Korea) were planted in 24-well trays filled with nursery soil (Bunong Horticulture Nursery Soil, Bunong, Korea). The trays were maintained at 25 ± 3 °C and a 77 ± 5% RH under a 12 h light/12 h dark photoperiod for four weeks. Subsequently, the seedlings were transferred into vinyl pots (7.5 cm in diameter, 6 cm in height). Seven and three days before inoculation, each plant was treated with 20 mL of soil drench and 5 mL of foliar spray per pot, using 500-fold, 1000-fold, 2000-fold, and 4000-fold dilutions of M21F004 CB medium or OA at concentrations of 0.1, 1, and 10 µg/mL. Each dilution was supplemented with 250 µg/mL of Tween 20. Subsequently, a 20 mL cell suspension of *R. solanacearum* SL341 (10^8^ CFU/mL in 10 mM MgCl_2_) was applied by soil drenching [6]. DS was assessed 10 days after inoculation using the following scale: 0 = no leaf symptoms; 1 = one leaf wilted; 2 = two or three leaves wilted; 3 = four leaves wilted; and 4 = plant death [50]. Sungbocycline, prepared at 1000 µg/mL in a Tween 20 solution, served as a positive control. Each experiment was conducted twice, with nine replicates.

#### 4.8.4. Soft Rot of Kimchi Cabbage

Kimchi cabbage seeds (cultivar ‘Chunkwang’; Sakada Korea, Seoul, Republic of Korea) were planted in 24-well trays containing nursery soil (Bunong Horticulture Nursery Soil, Bunong, Korea) and grown for three weeks. The seedlings were then transferred to vinyl pots (7.5 cm in diameter, 6 cm in height) 24 h before the experimental treatments began. Seven and three days before inoculation, plants were treated with a soil drench of 20 mL per pot using 500-fold, 1000-fold, 2000-fold, and 4000-fold dilutions of M21F004 CB medium or OA concentrations of 0.1, 1, and 10 µg/mL. Each dilution was supplemented with Tween 20 at a concentration of 250 µg/mL. Subsequently, the treated plants were inoculated with 20 mL of *P. carotovorum* subsp. *carotovorum* cell suspension (10^7^ CFU/mL, containing 10 mM MgCl_2_) applied as a soil drench [50]. The seedlings and treated plants were maintained at 25 ± 3 °C and 77 ± 5% RH under a 12 h light/12 h dark cycle. DS was evaluated 5 days after inoculation using a scale from 0 to 5 [51]: 0 = no symptoms; 1 = one or two pencil-line streaks; 2 = two pencil-line streaks; 3 = leaf chlorosis or bleaching; 4 = leaf necrosis; and 5 = plant death. Sungbocycline, prepared at 1000 µg/mL concentration, served as the positive control. Each experiment was conducted twice, with six replicates.

### 4.9. Calculation of the Control Value

The control value was calculated using the formula below:

(1) $$Control\;value\left(\%\right)=\frac{DS\;of\;control-DS\;of\;treatment}{DS\;of\;untreated\;control}\times 100$$

### 4.10. Statistical Analysis

All statistical data from in vitro and in vivo experiments were expressed as the mean ± standard deviation. Data were analyzed using one-way analysis of variance followed by Tukey’s post hoc test (*p* < 0.05), utilizing SPSS version 23.0 (SPSS Inc., Chicago, IL, USA).

## 5. Conclusions

This study identified and characterized the marine lactic acid bacterium *Pediococcus* sp. M21F004 as a promising biocontrol agent with broad-spectrum antimicrobial activity against bacterial and fungal phytopathogens. The strain’s active metabolite, OA, demonstrated significant in vitro and in vivo efficacy, including the suppression of diseases such as cucumber *Fusarium* wilt, creeping bentgrass dollar spot, tomato bacterial wilt, and kimchi cabbage soft rot. Notably, *Pediococcus* sp. M21F004 exhibited the ability to induce systemic resistance in plants, as evidenced by *PR1pro::GUS* expression in *Arabidopsis thaliana*, underscoring its dual function as both a direct antimicrobial agent and a resistance inducer.

These findings highlight *Pediococcus* sp. M21F004 as a sustainable alternative to chemical pesticides, offering eco-friendly solutions for plant disease management. The identification of OA as a primary active compound provides new insights into its mode of action against plant pathogens and broadens its potential applications in agriculture. Future studies should investigate the synergistic roles of other bioactive metabolites produced by *Pediococcus* sp. M21F004, optimize delivery systems for practical use, and assess field-scale efficacy to establish its role as a key component of integrated pest management strategies. This study represents a significant step toward advancing biopesticide development, addressing the urgent need for environmentally sustainable agricultural practices.

## Figures and Tables

**Table 1:** Marine bacterial strains used in this study.

No.	Strain	Identity	Similarity (%)	Sampling Site	Coordinates	Isolation Source
1	M19A1R17	*Rhodococcus kroppenstedtii*	98.47	Bieung, Gunsan-si, Jeollabuk-do	35°56′17.61″ N 126°31′52.49″ E	*Ulva* sp.
2	M19A1S3	*Rhodococcus trifolii*	98.31			
3	M19A1S10	*Rhodococcus trifolii*	98.31			
4	M19B1S5	*Algibacter lectus*	98.32	Gujwa-eup, Jeju-si, Jeju-do	33°31′30.66 N 126°51′41.43″ E	Seawater
5	M19B1Z5	*Pseudoalteromonas carrageenovora*	98.59			
6	M19B2R8	*Hydrogenophaga taeniospiralis*	98.55			*Undaria pinnatifida*
7	M19B2S3	*Colwellia agarivorans*	98.57			
8	M19B3S6	*Paraglaciecola aquimarina*	95.53			*Codium fragile*
9	M19C2D2	*Alteromonas marina*	97.08	Seongsan-eup, Seogwipo-si, Jeju-do	33°27′3.7″ N 126°55′27.06″ E	*Ulva* sp.
10	M19C2S11	*Alteromonas oceani*	98.07			
11	M19C2S14	*Octadecabacter algicola*	98.05			
12	M19C2Z2	*Psychrosphaera ytuae*	98.62			
13	M19C1D14	*Winogradskyella endarachnes*	99.59			
14	M19E1R33	*Wocania ichthyoenteri*	97.71	Yonghyeon-myeon, Sacheon-si, Gyeongsangnam-do	35°2′28.7″ N 128°2′23.2″ E	Seawater
15	M19E2S8	*Altererythrobacter rubellus*	99.49	Daebang-dong, Sacheon-si, Gyeongsangnam-do	34°55′43.5″ N 128°3′24.8″ E	
16	M19E3S8	*Marivita litorea*	97.92	Yonghyeon-myeon, Sacheon-si, Gyeongsangnam-do	35°0′34.2″ N 128°2′48.7″ E	
17	M20A1S7	*Gemmobacter fulvus*	97.62	Sinpyeong-myeon, Dangjin-si, Chungcheongnam-do	36°52′51″ N 126°49′39″ E	Seawater
18	M20A1S8	*Qipengyuania spongiae*	97.44			
19	M20A2S1	*Shewanella septentrionalis*	99.21	Seongmun-myeon, Dangjin-si, Chungcheongnam-do	37°0′13.1″ N 126°37′33.1″ E	Seawater
20	M20A5R6	*Janthinobacterium lividum*	99.66			*Ulva* sp.
21	M20A5R12	*Hydrogenophaga laconesensis*	98.48			
22	M20A5S7	*Nocardioides furvisabuli*	99.36			
23	M20A5S9	*Simplicispira limi*	99.66			
24	M20A3D2	*Aquimarina macrocephali*	97.30	Daesan-eup, Seosan-si, Chungcheongnam-do	36°58′3″ N 126°20′11.2″ E	Seawater
25	M20A3S3	*Dokdonia sinensis*	95.30			
26	M20A3S9	*Roseobacter insulae*	98.34			
27	M20A3S10	*Parerythrobacter lutipelagi*	97.20			
28	M20A3S11	*Cognatiyoonia koreensis*	97.11			
29	M20A3Z3	*Maribacter aestuarii*	97.03			
30	M20A8D8	*Motilimonas cestriensis*	99.79			Sediment
31	M20A8S13	*Hoppeia youngheungensis*	97.43			
32	M20A4R8	*Pseudomonas bohemica*	98.63	Sinpyeong-myeon, Dangjin-si, Chungcheongnam-do	36°52′47.8″ N 126°49′37.9″ E	*Rumex crispus*
33	M20A4R15	*Herminiimonas aquatilis*	100.00			
34	M20A4S4	*Neorhizobium tomejilense*	97.36			
35	M20B1D1	*Psychroflexus montanilacus*	98.32	Munnae-myeon, Haenam-gun, Jeollanam-do	34°35′45.2″ N 126°16′58.6″ E	Sediment
36	M20B1R5	*Halomonas johnsoniae*	98.28			
37	M20B1Z1	*Halomonas azerica*	98.63			
38	M20B1Z2	*Halomonas arcis*	98.79			
39	M20B5D3	*Halomonas azerica*	98.90			
40	M20B5D4	*Halomonas azerica*	98.49			
41	M20B5D5	*Halomonas azerica*	98.90			
42	M20B5D10	*Salegentibacter lacus*	99.72			
43	M20B5D12	*Salinimicrobium xinjiangense*	97.86			
44	M20C1R1	*Tenacibaculum mesophilum*	98.06	Byeonsan-myeon, Buan-gun, Jeollabuk-do	35°40′47.3″ N 126°31′48.4″ E	*Ulva* sp.
45	M20C3D11	*Microbulbifer rhizosphaerae*	97.97	Jinseo-myeon, Buan-gun, Jeollabuk-do	35°35′44.6″ N 126°37′2.4″ E	Sediment
46	M20D1D7	*Muricauda abyssi*	99.10	Iwon-myeon, Taean-gun, Chungcheongnam-do	36°57′47.5″ N 126°17′24.2″ E	Seawater
47	M21F001	*Latilactobacillus sakei subsp. sakei*	100.00	Chukbok-dong, Mokpo-si, Jeollanam-do	34°47′16.6″ N 126°23′23.6″ E	*Platichthys stellatus*
48	M21F003	*Kocuria salsicia*	99.86			
49	M21F004	*Pediococcus inopinatus*	100.00			
50	M21F006	*Rhodococcus qingshengii*	100.00			

**Table 2:** GC-MS analysis of detected possible compounds produced by M21F004.

Retention Time (min)	Area	Total Area (%)	Similarity (%)	Possible Compounds
8.52	24,560,882	16.2	85	3-Isobutylhexahydropyrrolo [1,2-a] pyrazine-1,4-dione
9.228	16,710,129	11.0	90	3-Isobutylhexahydropyrrolo [1,2-a] pyrazine-1,4-dione
9.345	24,933,274	16.5	93	3-Isobutylhexahydropyrrolo [1,2-a] pyrazine-1,4-dione
9.45	14,513,256	9.6	89	Palmitic acid
10.69	60,369,540	39.9	95	Oleic acid
12.35	10,369,682	6.9	92	3-Benzyl-hexahydro-pyrrolo [1,2-a] pyrazine-1,4-dione

GC-MS, gas chromatography–mass spectrometry.

## Data Availability

The data are contained within the article and Supplementary Materials.

## Supplementary Materials

The following supporting information can be downloaded at: https://www.mdpi.com/article/10.3390/md22120534/s1. Table S1: Antagonistic activity of 50 selected strains against plant pathogenic bacteria; Table S2: Antagonistic activity of 50 selected strains against plant pathogenic fungi; Figure S1: Detection of hexane extract and OA standard using GC-MS. OA, oleic acid; GC-MS, gas chromatography–mass spectrometry.
