# Supplementary material for: Exploring Pediococcus sp. M21F004 for Biocontrol of Bacterial and Fungal Phytopathogens
Source: Mar Drugs. 2024 Nov 28;22(12):534. doi: 10.3390/md22120534 (PMC11676420; doi:10.3390/md22120534)
Supplement: Supplementary file 1 [file marinedrugs-22-00534-s001.zip › marinedrugs-3305323-supplementary.pdf]

Supplementary material

Exploring *Pediococcus* sp. M21F004 for biocontrol of bacterial and fungal phytopathogens

Van Thi Nguyen[1,†], Yong Min Kwon[2,†], Ae Ran Park[3], Nan Hee Yu[3], Grace Choi[4,*], and Jin-Cheol Kim[1,3,*]

[1] Department of Agricultural Chemistry, Institute of Environmentally Friendly Agriculture, College of Agriculture and Life Sciences, Chonnam National University, Gwangju 61186, Republic of Korea

[2] Department of Biological Application and Technology, National Marine Biodiversity Institute of Korea, Seocheon 33662, Republic of Korea

[3] Plant Healthcare Research Institute, JAN153 Biotech Incorporated, Jeongeup 56212, Republic of Korea

[4] Department of Biomaterial Research, National Marine Biodiversity Institute of Korea, Seocheon 33662, Republic of Korea

*Correspondences: gchoi@mabik.re.kr; Tel.: +82-41-950-0930 (G.C), kjinc@jnu.ac.kr; Tel.: +82-62-530-2132 (J.-C.K)

†These authors contributed equally to this work.

Table S1. Antagonistic activity of 50 selected strains against plant pathogenic bacteria.

No.	Strains code	Pathogenic bacteria (MIC, %) *			
		Agrobacterium tumefaciens	*Pectobacterium carotovora* subsp. *Carotovora*	*Ralstonia solanacearum*	*Xanthomonas arboricola* pv. *pruni*
1	M19A1R17	-	-	-	-
2	M19A1S3	-	-	-	-
3	M19A1S10	-	-	-	-
4	M19B1S5	-	-	-	-
5	M19B1Z5	-	-	-	-
6	M19B2R8	-	-	-	-
7	M19B2S3	-	-	-	-
8	M19B3S6	-	-	-	-
9	M19C2D2	-	-	-	-
10	M19C2S11	-	-	-	-
11	M19C2S14	-	-	-	-
12	M19C2Z2	-	-	-	-
13	M19C1D14	-	-	-	-
14	M19E1R33	-	-	-	-
15	M19E2S8	-	-	-	-
16	M19E3S8	-	-	-	-
17	M20A1S7	-	-	-	-
18	M20A1S8	-	-	-	-
19	M20A2S1	-	-	-	-
20	M20A5R6	10	-	-	-
21	M20A5R12	-	-	-	-
22	M20A5S7	-	-	-	-
23	M20A5S9	-	-	-	-
24	M20A3D2	-	-	-	-
25	M20A3S3	-	-	-	-

No.	Strains code	Pathogenic bacteria (MIC, %) *			
		Agrobacterium tumefaciens	*Pectobacterium carotovora* subsp. *carotovora*	*Ralstonia solanacearum*	*Xanthomonas arboricola* pv. *pruni*
26	M20A3S9	-	-	-	-
27	M20A3S10	-	-	-	-
28	M20A3S11	-	-	-	-
29	M20A3Z3	-	-	-	-
30	M20A8D8	-	-	-	-
31	M20A8S13	-	-	-	-
32	M20A4R8	-	-	-	-
33	M20A4R15	-	-	-	-
34	M20A4S4	-	-	-	-
35	M20B1D1	-	-	-	-
36	M20B1R5	-	-	-	-
37	M20B1Z1	-	-	-	-
38	M20B1Z2	-	-	-	-
39	M20B5D3	-	-	-	-
40	M20B5D4	-	-	-	-
41	M20B5D5	-	-	-	-
42	M20B5D10	-	-	-	-
43	M20B5D12	-	-	-	-
44	M20C1R1	-	-	-	-
45	M20C3D11	-	-	-	-
46	M20D1D7	-	-	-	-
47	M21F001	-	-	-	10
48	M21F003	-	-	-	-
49	M21F004	10	-	10	10
50	M21F006	-	-	-	-

*The minimum inhibitory concentration (MIC) values represent the percentage of cell-free supernatant (CFS) volume from *Pediococcus* sp. M21F004 relative to the total assay volume used to suppress the growth of the specified pathogens. For example, an MIC of 10% indicates that 10 μL of CFS was added to 90 μL of assay medium, resulting in a total volume of 100 μL.

Table S2. Antagonistic activity of 50 selected strains against plant pathogenic fungi.

No.	Strains code	Pathogenic fungi (MIC, %) *			
		Phytophthora infestans	*Rhizoctonia solani*	*Clarireedia homoeocarpa*	*Pythium aphanidermatum*
1	M19A1R17	-	-	-	-
2	M19A1S3	-	-	-	-
3	M19A1S10	-	-	-	-
4	M19B1S5	-	-	-	-
5	M19B1Z5	-	-	-	-
6	M19B2R8	-	-	-	-
7	M19B2S3	-	-	-	-
8	M19B3S6	-	-	-	-
9	M19C2D2	-	-	-	-
10	M19C2S11	-	-	-	-
11	M19C2S14	-	-	-	-
12	M19C2Z2	-	-	-	-
13	M19C1D14	-	-	-	-
14	M19E1R33	-	-	-	-
15	M19E2S8	-	-	-	-
16	M19E3S8	-	-	-	-
17	M20A1S7	-	-	-	-
18	M20A1S8	-	-	-	-
19	M20A2S1	-	-	-	-
20	M20A5R6	-	-	-	5
21	M20A5R12	-	-	-	-
22	M20A5S7	-	-	-	-
23	M20A5S9	-	-	-	
24	M20A3D2	-	-	-	-
25	M20A3S3	-	-	-	-

No.	Strains code	Pathogenic fungi (MIC, %) *			
		Phytophthora infestans	*Rhizoctonia solani*	*Clarireedia homoeocarpa*	*Pythium aphanidermatum*
26	M20A3S9	-	-	-	-
27	M20A3S10	-	-	-	-
28	M20A3S11	-	-	-	-
29	M20A3Z3	-	-	-	-
30	M20A8D8	-	-	-	-
31	M20A8S13	-	-	-	-
32	M20A4R8	-	-	-	-
33	M20A4R15	-	-	-	-
34	M20A4S4	-	-	-	-
35	M20B1D1	-	-	-	-
36	M20B1R5	-	-	-	-
37	M20B1Z1	-	-	-	-
38	M20B1Z2	-	-	-	-
39	M20B5D3	-	-	-	-
40	M20B5D4	-	-	-	-
41	M20B5D5	-	-	-	-
42	M20B5D10	-	-	-	-
43	M20B5D12	-	-	-	-
44	M20C1R1	-	-	-	-
45	M20C3D11	-	-	-	5
46	M20D1D7	-	-	-	-
47	M21F001	10	10	10	-
48	M21F003	10	10	10	
49	M21F004	10	10	5	-
50	M21F006	-	-	-	-

*The minimum inhibitory concentration (MIC) values represent the percentage of the cell-free supernatant (CFS) volume from *Pediococcus* sp. M21F004 relative to the total assay volume used to suppress the growth of the specified pathogens. For example, an MIC of 10% indicates that 10 μL of CFS was added to 90 μL of assay medium, resulting in a total volume of 100 μL.

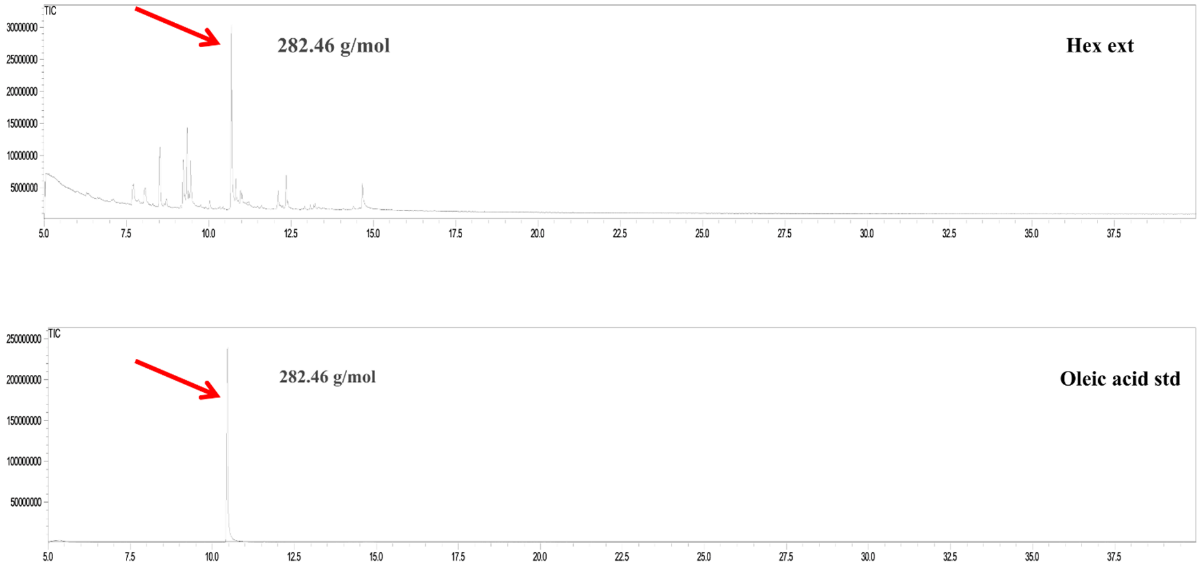

Figure S1. Detection of hexane extract and OA standard using GC-MS. OA, oleic acid; GC-MS, Gas Chromatography-Mass Spectrometry.
